# Indigenous early warning indicators for improving natural hazard predictions

**DOI:** 10.4102/jamba.v17i1.1754

**Published:** 2025-04-04

**Authors:** Masego M. Motsumi, Livhuwani D. Nemakonde

**Affiliations:** 1 African Centre for Disaster Studies, Unit for Environmental Science and Management, Faculty of Natural and Agricultural Sciences, North-West University, Potchefstroom, South Africa

**Keywords:** natural hazards, Indigenous knowledge (IK), indigenous early warning indicators, seasonal climate and weather forecast, disasters, complementary

## Abstract

Indigenous knowledge plays a crucial role in predicting hazardous events, particularly for rural communities who are not reached by conventional early warning systems. Historical knowledge of hazard occurrence stored in the repository of these communities combined with their ability to observe environmental indicators, enhances their preparedness for, response to, and recovery from disasters. This study sought to gain insights into the Indigenous indicators used by rural communities in the Joe Morolong Local Municipality, Northern Cape, South Africa, to predict natural hazards, and explores how these indicators could complement meteorological seasonal climate and weather forecasts. The study was conducted in Joe Morolong Local Municipality, Northern Cape, South Africa. A phenomenological approach, which is a form of qualitative research design, was employed, with data collected through focus group discussions and virtual interviews with key informants, involving 109 participants. The findings reveal that communities rely on various environmental signs, including changes in vegetation, lunar cycles, cloud formations, bird behaviors, and wind speed and direction, to predict and anticipate hazardous events. These traditional methods, refined over generations, provide localised, trusted, and contextually relevant early warning systems that enhance disaster preparedness. By recognising and integrating these Indigenous indicators with meteorological forecasts, disaster risk management efforts can be strengthened, ensuring that rural communities have more comprehensive and effective tools for mitigating the risks and impacts of natural hazards.

**Contribution:**

This study emphasises the value of Indigenous knowledge as a vital resource for enhancing disaster and climate resilience, as well as improving early warning systems.

## Introduction

Western scientific knowledge and Indigenous knowledge (IK) are often positioned as distinct and competing systems, a divide deeply rooted in their respective epistemological foundations (Briggs 2005). Briggs (2005) argues that the tensions created by the binary divide between Western science and IK clearly persist despite many well-intentioned efforts to reduce or eliminate them. Some scholars such as Le Grange (2004) challenge this binary perspective, questioning whether these knowledge systems should be seen as competing frameworks or complementary perspectives. Agrawal (1995) further argues that IK is not merely an alternative or opposing system to Western knowledge (WK) but serves to bridge cultural and conceptual gaps in Western epistemology. Despite numerous efforts to dissolve this divide, tensions persist (Briggs 2005). Agrawal (1995) even suggests that if it were possible to revisit historical discourse, terms such as ‘Indigenous’ and ‘scientific’ should be abandoned, as knowledge should be evaluated based on its relevance, empirical testability and social utility rather than its classification. Despite the challenges in integrating these knowledge systems (Johnson et al. 2016), it is widely acknowledged that their complementarity can offer new perspectives and approaches to existing challenges.

**FIGURE 1:**
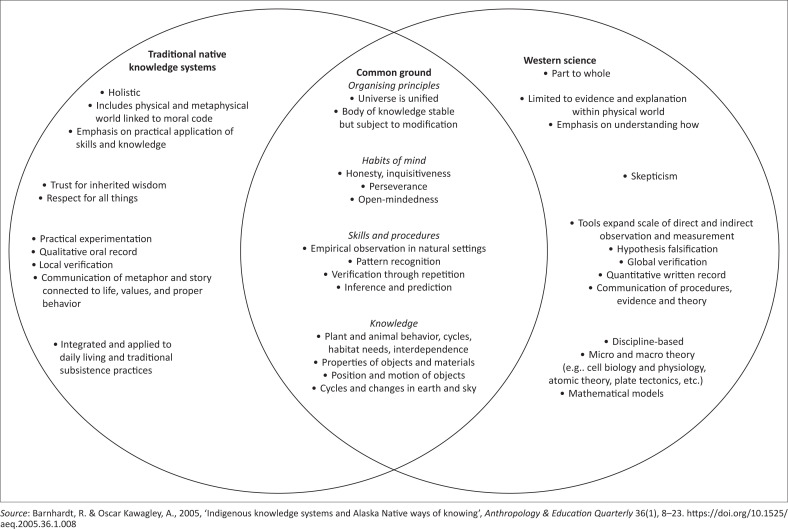
Qualities associated with traditional (Indigenous) knowledge systems and Western science.

In Figure 1, Barnhardt and Kawagley (2005) have demonstrated that despite several differences, there are many shared tenets when the nature of contemporary science and the nature of IK are compared. Both contemporary science and IK are empirical (although IK also has a metaphysical component), both are tentative (and subjected to change), both are inferential, both are creative and both are socially and culturally based (De Beer & Petersen 2016). Yeh (2016) emphasises that while epistemological differences will remain, fostering appreciation for diverse ways of knowing can lead to more productive applications of knowledge.

Indigenous people have their own ways of looking at and relating to the world, the universe and each other (Ascher 2018). As Maweu (2011) puts it, for many traditional communities, IK forms a holistic worldview, which is inseparable from their very ways of life – their cultural values, spiritual beliefs and customary legal systems. Their traditional education processes are carefully constructed around observing natural processes, adapting modes of survival, obtaining sustenance from the plant and animal world and using natural materials to make their tools and implements (Barnhardt & Kawagley 2005). Many indigenous communities already employ scientific principles in their daily lives, incorporating empirical observations into subsistence activities (Barnhardt & Kawagley 2005; Durie 2004). Lavallée (2009) describes indigenous science as a knowledge system shaped through revelation, traditional teachings and long-term empirical observations in real-world settings. Nakashima, Prott and Bridgewater (2000) conceptualise IK as a sophisticated system encompassing a broad array of insights, including agriculture, hunting, fishing, animal husbandry and disease management. Indigenous knowledge is not only practical but also integral to cultural identity, spiritual beliefs and customary laws (Maweu 2011).

Indigenous communities have long studied environmental patterns, developing knowledge systems in meteorology, pharmacology, earth sciences and ecology (Barnhardt & Kawagley 2005; Burgess 1999). Indigenous knowledge allows indigenous communities to monitor seasonal changes and recognise early warning signs of disasters (Dekens 2007). Therefore, it plays a crucial role in disaster risk reduction (DRR), providing localised, context-specific understandings of hazards that are often overlooked by scientific models (Bwambale et al. 2022). According to the Sendai Framework for DRR, IK is essential for enhancing disaster preparedness, resilience and response strategies, emphasising its role as a complement to scientific knowledge (Bwambale et al. 2022). Dekens (2007) outlines four critical aspects of local knowledge for disaster preparedness: the ability to observe environmental surroundings, anticipate hazards through natural indicators, develop adaptation strategies and effectively communicate risks across generations. Observations of atmospheric conditions (e.g. wind), celestial elements (e.g. the sun and moon), animal behaviour (e.g. ants) and plant cycles (e.g. trees) have been used for centuries as early warning systems (Chang’a, Yanda & Ngana 2010; Chanza & Mafongova 2017).

The binary that has been created between indigenous and scientific knowledge in general is also seen in DRR, where technology is often associated with scientific knowledge (Balay-As, Marlowe & Gaillard 2018). As such, IK remains underutilised in formal disaster management strategies. Plotz, Chambers and Finn (2017) note that local communities often rely on traditional knowledge (TK) for climate forecasts rather than contemporary scientific models, partly because of accessibility issues and a lack of trust in external forecasting systems. However, acknowledging and incorporating traditional forecasting methods into scientific models can enhance the accuracy, reliability and acceptance of climate and weather predictions (Plotz et al. 2017). Hiwasaki, Luna and Shaw (2014) highlight that integrating both traditional and scientific knowledge systems strengthens community resilience. A well-documented case is the 1999 tsunami in Vanuatu, where early warnings were issued using both indigenous and scientific methods. Research showed that while both contributed to disaster response, IK played a more significant role in community preparedness and action (Walshe & Nunn 2012). When complemented by scientific advancements, IK can provide communities with a strong foundation for environmental decision-making (Hiwasaki et al. 2014).

Indigenous communities are often among the most vulnerable to natural disasters (Whitfield et al. 2015); yet, their local knowledge remains largely underutilised in DRR and climate adaptation strategies (Makondo & Thomas 2018). This study sought to gain insights into the indigenous indicators used by rural communities in the Joe Morolong Local Municipality, Northern Cape, South Africa, to predict natural hazards and explores how these indicators could complement meteorological seasonal climate and weather forecasts. The study highlights how local communities use indigenous indicators – such as changes in vegetation, animal behaviour, celestial patterns and wind direction – to predict hazardous events. These methods, refined over generations, serve as localised and trusted early warning systems that enhance community preparedness. Rather than advocating for the integration of knowledge systems, which implies the assimilation of one into the other, this study supports the complementary and simultaneous use of indigenous and Western scientific knowledge. The term ‘integration’ often carries connotations of dominance, where one knowledge system is subsumed by another. In contrast, complementarity allows for mutual enrichment and the coexistence of diverse epistemologies. This perspective aligns with previous studies that have emphasised the validity and utility of traditional climate forecasting methods (Alessa et al. 2016; Chang’a et al. 2010; Lefale 2010; Masinde 2014; Nanja in Ajayi & Mafongoya 2017; Zuma-Netshiukhwi, Stigter & Walker 2013). However, this study is the first to specifically document traditional forecasting methods in the Northern Cape, South Africa.

Following this introduction, the article details the research methods employed, including qualitative data collection through focus group discussions (FGDs) and virtual interviews with key informants (KIs). The findings section presents the indigenous forecasting indicators identified by participants, analysing their effectiveness and reliability. The discussion then situates these findings within the broader discourse on DRR, emphasising the potential of complementary knowledge systems to enhance disaster preparedness. Finally, the article concludes with recommendations for incorporating IK into DRR policies and practices, ensuring that traditional wisdom continues to play a vital role in safeguarding communities against natural hazards.

## Research methods and design

This study is premised in pragmatism and employs a phenomenological approach. Pragmatism, as described by Saunders et al. (2019), holds that concepts are meaningful only when they contribute to action, starting with problem identification and aiming to provide practical solutions for future practice. Because the study sought real-life insights and experiences regarding IK indicators that could complement meteorological seasonal climate and weather forecasts, a phenomenological approach was chosen. This approach is ideal for exploring participants’ lived experiences, as it delves into how individuals interpret and give meaning to their existence (Frechette et al. 2020).

### Sampling methods and data collection

Data were collected from the communities in the five rural villages of Bendell, Bothithong, Dithakong, Gasese and Tsineng in the Morolong Local Municipality, Northern Cape. A total of 100 participants participated in the study through FGDs, while 9 KIs were interviewed via virtual platforms. Virtual platform was employed because it provided better flexibility and ease of data collection when compared to the traditional strategy of visiting KIs (Archibald et al. 2019). Focus group discussions align with indigenous research methods by fostering rich, interactive discussions that incorporate interpretations of parables, songs, spiritual symbols and talking circles – key elements of indigenous epistemology. This method encouraged openness and trust among participants, facilitating in-depth conversations while upholding the indigenous value of relationships. While broad thematic questions guided the discussions, the researcher continuously reflected on their positionality, ensuring external interpretations were not imposed on the data. Key informants were carefully chosen based on their field of expertise in line with the research topic. They included one representative each from various private and governmental institutions, such as the Tribal authority – Chief from Bahurutshe Traditional House (1), the Community chairperson of Tsineng Community Integrated Development Structure responsible for the sampled villages (1); An Induna (1), National Disaster Management Centre (NDMC) (1); Provincial Disaster Management Centre (PDMC) (1); District Disaster Management Centre (DDMC) (1); Municipal IDP personnel (1) and Independent facilitator personnel for Indigenous Knowledge Systems (IKS) in the rural community (1) and an Independent Facilitator in Community and Organisation Development (1).

### Data analysis

The collected data were analysed using content or thematic analysis, which involves organising the data into themes (Guest, MacQueen & Namey 2012) to transform the subject matter into easily understandable insights (Akinyode & Khan 2018). Thematic analysis was chosen for this research because it effectively captures participants’ views, opinions, experiences, knowledge and values. It also aligns with the narrative and storytelling format of FGDs, allowing indigenous voices and knowledge to be respected and preserved. To maintain authenticity, participants’ direct quotes were recorded in Setswana, the primary language spoken in the study area and then loosely translated into English for readability in this article. This method ensured that the participants’ original voices remained central to the analysis. Following the approach outlined by DiCicco-Bloom and Crabtree (2006), the analysis was conducted concurrently with data collection to enhance the understanding of the themes under investigation. Each FGD participant is assigned a unique code, such as Bend1F or Both1M (see Table 1), which captures key details about their identity while ensuring anonymity. The code systematically represents the participant’s village, focus group, assigned number, and gender, allowing for clear categorisation. For Key Informants (see Table 2), the codes indicate the institution and/or identifier, gender, and assigned number. This coding system is essential for maintaining confidentiality while facilitating the organisation and analysis of interview data. It enables the researcher to accurately present participants’ insights and directly quote their responses without compromising their privacy. Table 3 outlines the main themes and sub-themes that emerged from the study and formed the basis for analysing the data.

**TABLE 1:** FGD Participants’ codes.

Village Name	FGD1			FGD2			FGD3			FGD4		
	Gender	Number	Code	Gender	Number	Code	Gender	Number	Code	Gender	Number	Code
Bendell	Female	1	Bend1F1	Female	3	Bend2F3	Male	8	Bend3M8	Female	4	Bend4F4
	Female	2	Bend1F2	Male	4	Bend2M4	Male	9	Bend3M9	Female	5	Bend4F5
	Male	1	Bend1M1	Male	5	Bend2M5	Male	10	Bend3M10	Male	13	Bend4M13
	Male	2	Bend1M2	Male	6	Bend2M6	Male	11	Bend3M11	Male	14	Bend4M14
	Male	3	Bend1M3	Male	7	Bend2M7	Male	12	Bend3M12	Male	15	Bend4M15
Bothithong	Female	6	Both1F6	Male	20	Both2M20	Female	7	Both3F7	Female	9	Both4F9
	Male	16	Both1M16	Male	21	Both2M21	Female	8	Both3F8	Female	10	Both4F10
	Male	17	Both1M17	Male	22	Both2M22	Male	25	Both3M25	Female	11	Both4F11
	Male	18	Both1M18	Male	23	Both2M23	Male	26	Both3M26	Male	28	Both4M28
	Male	19	Both1M19	Male	24	Both2M24	Male	27	Both3M27	Male	29	Both4M29
Dithakong	Male	30	Dith1M30	Male	35	Dith2M35	Female	12	Dith3F12	Female	14	Dith4F14
	Male	31	Dith1M31	Male	36	Dith2M36	Female	13	Dith3F13	Male	43	Dith4M43
	Male	32	Dith1M32	Male	37	Dith2M37	Male	40	Dith3M40	Male	44	Dith4M44
	Male	33	Dith1M33	Male	38	Dith2M38	Male	41	Dith3M41	Male	45	Dith4M45
	Male	34	Dith1M34	Male	39	Dith2M39	Male	42	Dith3M42	Male	46	Dith4M46
Gasese	Female	15	Gase1F15	Female	16	Gase2F16	Male	54	Gase3M54	Female	18	Gase4F18
	Male	47	Gase1M47	Female	17	Gase2F17	Male	55	Gase3M55	Female	19	Gase4F19
	Male	48	Gase1M48	Male	51	Gase2M51	Male	56	Gase3M56	Male	59	Gase4M59
	Male	49	Gase1M49	Male	52	Gase2M52	Male	57	Gase3M57	Male	60	Gase4M60
	Male	50	Gase1M50	Male	53	Gase2M53	Male	58	Gase3M58	Male	61	Gase4M61
Tsineng	Female	20	Tsine1F20	Female	21	Tsine2F21	Male	70	Tsine3M70	Male	75	Tsine4M75
	Male	62	Tsine1M62	Male	66	Tsine2M66	Male	71	Tsine3M71	Male	76	Tsine4M76
	Male	63	Tsine1M63	Male	67	Tsine2M67	Male	72	Tsine3M72	Male	77	Tsine4M77
	Male	64	Tsine1M64	Male	68	Tsine2M68	Male	73	Tsine3M73	Male	78	Tsine4M78
	Male	65	Tsine1M65	Male	69	Tsine2M69	Male	74	Tsine3M74	Male	79	Tsine4M79

**TABLE 2:** Key Informant codes.

Institution/Identifier	Gender	Number	Code
Chief from Bahurutshe Traditional House	Male	1	CHIEFM1
Tsineng Community Integrated Development Chairperson	Male	2	CHATSIM2
Induna	Male	3	INDM3
National Disaster Management Centre	Male	4	NDMCM4
Provincial Disaster Management Centre	Male	5	PDMCM5
District Disaster Management Centre	Male	6	DDMCM6
Municipal IDP Personnel	Female	7	MunIDP F7
IKS independent Facilitator	Female	8	IKSIFF8
Community and Organisational Development Facilitator	Female	9	CODFM9

**TABLE 3:** Themes and sub-themes used for presenting the findings.

Themes	Sub-themes
1: Participants’ understanding of indigenous and Western knowledge and complementary use of both	1.1: Understanding of IK within local communities 1.2: Understanding of WK-based approaches to disaster risk reduction within local communities 1.3: Understanding the need for complementary use of IKS and WKS in DRR
2: Indigenous indicators used to forecast or predict the different hazards and disasters in the study sites	2.1: Birds indicators 2.2: Plants, shrubs and herbs indicators 2.3: Lunar, climatic and wind indicators 2.4: Environmental and Indigenous Belief Systems indicators

IK, indigenous knowledge; WK, Western knowledge; IKS, Indigenous Knowledge Systems; WKS, Western Knowledge Systems; DRR, Disaster Risk Reduction.

### Ethical considerations

This work is a component of a larger study ‘A Framework to Integrate Indigenous Knowledge into Disaster Risk Reduction for Building Disaster Resilience in the Northern Cape, South Africa’ by the main author. Ethical clearance to conduct this study was obtained from the North-West University Faculty of Natural and Agricultural Sciences Ethics Committee (FNAS REC) (No. NWU-01721-20-A9). Cultural protocols and traditions of the participating communities were carefully respected with permission to conduct the research sought from the Tribal Authorities of the identified villages, ensuring alignment with local governance structures. Before starting the research, verbal free, and informed consent was obtained from the participants, who were assured of confidentiality and anonymity to protect their rights. During data collection, the researcher adhered to strict confidentiality and anonymity protocols, ensuring that no deceptive methods were used to influence participants. This approach helped build a long-term, trust-based relationship with the communities, demonstrating a commitment to ethical research practices and cultural sensitivity. By honouring community traditions and prioritising participant well-being, the study fostered a collaborative environment based on mutual respect and understanding.

## Results

### Findings and analysis

This section presents the overall findings of the study, derived from both the FGDs and KI interviews.

### Participants’ understanding of indigenous and Western Knowledge and complementary use of both in disaster risk reduction

The findings of the study reveal that participants in both FGDs, and KI interviews have a thorough understanding of IKS, the Western Knowledge Systems (WKS) and the need for complementary use of both in DRR. The findings of this theme are presented under three different sub-themes.

#### Participants’ understanding of Indigenous knowledge

The findings of the study reveal a deep understanding and appreciation of IK among participants in both FGDs and KI interviews. Across the groups, participants consistently emphasised that IK is an integral part of their daily lives and is not something learned from books but rather acquired through lived experiences and oral traditions, highlighting the essential role of storytelling in its transmission.

Participants from the FGDs provided rich descriptions of how IK is embedded in their cultural and practical existence. One participant at Bothithong village captured this sentiment succinctly, stating: ‘Maele a re a rutilweng ke batsadi ba rona go amana ka tsela ya botshelo’ (Bothithong, FGD1, Both1M16), which loosely translate to: ‘Information taught by our parents on survival and the way of life within the village’. Another participant at Gasese village elaborated on the practical applications of IK, explaining:

‘E ke thuto e e re bontshang gore re dire dilo ka tsela e ntseng jang jaaka re rutilwe ke batsadi ba rona gore re kgotlhelele mo botshelong, thata thata go akaretsa temo-thuo. Gape ba re rutile go nna le kitso ya ponelo pele ya loapi gore diphologolo tsa go tshwana le dinku, dipudi, dkgomo le bo mmutlwa di tlhajwa leng.’ (Gasese, FGD3, Gase3M55)

Which loosely translates to:

‘This is the knowledge on how we do things as taught by our parents back in the days for us to live and survive, such as knowledge on farming and planting. They also taught us the ability to predict rain and knowledge on the slaughtering of animals such as lamb, goats, cows, and even rabbits.’

These reflections illustrate how IK is deeply intertwined with agricultural practices, environmental observation and traditional skills that ensure self-sufficiency.

A recurring concern among participants was the erosion of IK in modern times. Several participants lamented that younger generations are becoming increasingly disconnected from these traditional ways of knowing and doing things. They stressed that IK is invaluable and must be preserved, given that it is not formally documented and not taught in schools but rather passed down through oral traditions. The notion that: ‘[*M*]ost modern-day children are lost because they do not value indigenous practices’ (Dithakong, FGD2, Dith2M39), underscores a broader fear that the transmission of this knowledge may be disrupted by changing societal norms and influences.

Similarly, KIs demonstrated a profound understanding of IK, reinforcing its significance in shaping sustainable livelihoods and disaster resilience. One KI explained IK as:

‘[*T*]he body of knowledge passed on by the older generation of how things have been done, such as farming practices, traditional means to respond to disasters, and knowledge about how to protect and preserve livelihoods. IK is the know-how to do things and survive within the environment one is placed in.’ (KI, Participant 1, ChiefM1)

This perspective underscores the adaptability of IK, portraying it as a dynamic system of knowledge that enables communities to navigate environmental challenges and sustain their way of life. Another KI emphasised the evolving nature of IK, noting:

‘Systems that have been passed on from generation to generation. IK is part and parcel of who we are, and it is subject to also changes that have happened over the century.’ (KI, Participant 5, PDMCM5)

This statement highlights the fluidity of IK, acknowledging that while it is rooted in tradition, it also evolves in response to contemporary realities.

Overall, the findings suggest that both FGD participants and KIs recognise the indispensable role of IK in their communities. They regard it as a crucial resource for survival, cultural continuity and environmental stewardship. However, concerns over the diminishing transmission of IK to younger generations signal an urgent need for concerted efforts to safeguard and revitalise these knowledge systems. The deep respect for IK expressed by participants affirms its value, not only as a historical legacy but as a living, adaptive system that continues to shape their understanding of the world.

#### Participants’ understanding of Western knowledge-based approaches to disaster risk reduction

The findings reveal that participants in both the FGDs and KI interviews have a clear understanding of WK-based approaches, particularly in the context of DRR. In general, participants associated WK with modern advancements such as medicine, technology and sophisticated forecasting techniques, including those used in television and telephone weather predictions. Their perceptions reflected an awareness of WK as a system that introduces new ways of doing things, often in contrast to indigenous methods.

One participants from Tsineng Village, for instance, described WK as: ‘Kitso ya segopieno jaaka temo, temogo le go tsala ga leruo jaaka dikgomo le go lemoga metsamao yotlhe ya tsona’, (Tsineng, FGD4, Tsine4M76), which loosely translates to: ‘Western knowledge refers to Western ways of doing things, such as farming sensors that detect when a cow is about to give birth and track the location of cattle’. This perspective highlights an appreciation for the technological advancements that improve agricultural practices, indicating that local communities acknowledge the value of WK in enhancing efficiency and productivity. Similarly, another participant from Bendell Village referred to WK as: ‘Dilo tsa sesha tsa go dira dilo’, (Bendell, FGD1, Bend1F1), which loosely translates to: ‘[*N*]ewer ways to do things’. This simplified yet profound interpretation captures the essence of WK as an evolving body of knowledge that brings about change and innovation.

Despite their recognition of its benefits, many participants expressed concerns regarding the accessibility and applicability of WK-based DRR initiatives. While they acknowledged that these approaches bring valuable technical solutions, they also highlighted significant challenges in their implementation. A recurring theme among participants was that many DRR projects and technical interventions fail because of a lack of understanding within local communities and the high costs associated with maintaining the introduced technologies. For many, WK remains an external system that is not fully integrated into their daily lives. Language barriers and difficulties in comprehending the operation and purpose of modern technologies further complicate the effective utilisation of these interventions. As one Tsineng participant noted: ‘Re neelwa diteginologi tse gantsi re sa di tlhaloganyeng, mme e bile go sena tshedimosetso ya tiriso ya tsone’ (Tsineng, FGD2, Tsine2F21), which loosely translates to: ‘We are given technologies that we do not always understand, and there is no proper guidance on how we should use them’.

Key informants further contextualised WK within historical and global frameworks, viewing it as a system that gained prominence during the colonial era and subsequently became the dominant paradigm for organising various aspects of life worldwide. One KI characterised it as: ‘[*A*] more complex way of living and operating, with unique technical systems for presenting meteorological conditions and, eventually, legal standards and protocols for identifying who is eligible for financial help’ (KI, Participant 9, CODFM9). This suggests that WK is perceived not only as a technological advancement but also as a system that establishes structures for governance, regulation and resource allocation.

Key informants also emphasised that while Western DRR approaches have the potential to sustain communities and minimise risks, their effectiveness depends largely on proper education and awareness. One KI observed that: ‘[*T*]here is little time for consultations due to project time frames’ (KI, Participant 8, IKSIFF8), meaning that while DRR projects are designed to help, they often do not accommodate the time and effort required for proper community engagement and training. This lack of engagement results in limited participation, particularly among older indigenous individuals who may find it challenging to adapt to new technologies. Another informant explained:

‘Implementation normally goes well with younger people since there is quicker understanding, but this is not always the case when working with indigenous people, particularly when it comes to technology and the products offered to them.’ (KI, Participant 3, IndM3)

This statement highlights generational differences in the adoption of WK, with younger individuals showing greater adaptability, whereas older generations may struggle with unfamiliar systems.

Overall, the findings demonstrate that while local communities recognise the value of WK and its contributions to DRR, significant challenges hinder its full adoption. Issues such as language barriers, inadequate training, high costs and limited community involvement in decision-making processes create obstacles to successful implementation. The study underscores the need for more inclusive approaches that combine Western scientific advancements with IKS, ensuring that DRR initiatives are both effective and culturally relevant.

#### Participants’ understanding of the need for complementary use of Indigenous Knowledge Systems and Western Knowledge Systems in Disaster Risk Reduction

The findings reveal that participants in the FGDs demonstrated a deep understanding of DRR and were eager to contribute their knowledge. They expressed a strong desire for an integrated system that acknowledges and incorporates their IK alongside Western scientific approaches. According to participants, this integration would enhance preparedness, facilitate faster response times and provide more effective support during crises. Many believed that combining the two knowledge systems could also help reduce poverty through employment opportunities generated by DRR initiatives. In addition, some participants highlighted the potential benefits for the elderly in their communities, who are often the most vulnerable during disasters.

One participant from Tsineng Village emphasised the importance of unity in addressing community challenges, explaining:

‘Tirisano mmogo ya ditsamaiso tse pedi tse, e tla thusa go fedisa tsela ya go sa ipaakanyetsa maemo a tshoganyetso mme e re neye tshono e e botoka ya go ipaakanyetsa maemo a tshoganyetso le matshosetsi ka nako. Re umaka se ka ntlha ya gore ba setshaba sa legae ga ba na tirisano mmogo mo tharabolong ya mathata a selegae. Mme e bile ba ba nang le kitso ya setso ba utlwa botlhoko fa ba sa akaretswe mo ditsamaisong. Sakai, fa go na le kgathelelo ya diphefo tsa setsuatsuwe, setshaba sa selegae ba tshwanela ke go lekola maemo a dintlo tsa bagolo tse di weleng, mme fa go ka ne go nnile le go ipaakanyetsa ka nako ditlamorago tse dibotlhoko di ka bo thibetswe ka nako.’ (Tsineng, FGD4, Tsine4M78)

Which loosely translates to:

‘Complementary usage of both systems will eliminate existing reactionary mechanisms in the community, allowing us to be better prepared and predict our threats. We are saying this because we are not united in approaching community problems, and those who rely on IK are concerned if they are excluded. For example, during extreme winds, the community had to check on the elders of houses that collapsed, and if there had been an intervention before, it could have been avoided. This statement underscores the need for a proactive, rather than reactive, approach to disaster management, where IK is not sidelined but rather used to strengthen DRR strategies’.

Similarly, another participant from Gasese Village pointed out that integrating both knowledge systems would be particularly beneficial for poorer members of the community who lack the means to withstand severe hazardous events and stated:

‘Tirisano mmogo e thusa go nolofatsa le go fokotsa mathata a tshoganyetso ka ntlha ya gore ba kobo dikhutswane kgotsa bahumanegi ke bone gantsi ba amegang thata mo maemong a tshoganyetso.’ (Gasese, FGD4, Gase4F19)

Which loosely translates to:

‘Complementary usage will make it simpler for us to deal with disasters because the poor are often the most impacted and choose to give up’.

This highlights the disproportionate impact of disasters on economically disadvantaged individuals and the potential for knowledge integration to improve resilience.

Another crucial point raised by participants from Tsineng was the need for education and awareness to facilitate the successful implementation of DRR efforts. The participant noted:

‘Re dumela gore thuto le boitemogelo di botlhokwa thata ka ntlha ya gore di a tlhokafala mo setshabeng sa selegae. Mme se se tla thusa go thibela go sa tllhaloganyane fa ntse go amogelwa maiteko kgotsa lotseno lwa DRR go tokafatsa matshelo, go ipaakanyetsa mathata a tshoganyetso le go neelana ka tsa pholo.’ (Tsineng, FGD1, Tsine1M62)

Which loosely translates to:

‘We believe education and awareness are important since they are lacking in the community, as well as to avoid misunderstandings while receiving DRR efforts that improve lives, prepare for hazards, and provide healthcare options.’

Their concern suggests that the effectiveness of DRR interventions is often hindered by a lack of proper communication and understanding within the community. Participants recognised that without adequate education on how to utilise DRR initiatives, the full benefits of scientific and technological advancements may not be realised.

Furthermore, participants strongly believed that once IKS and WKS are properly integrated, communities would be better equipped to tackle climate change and mitigate its impacts. They stressed that those living in rural areas possess extensive knowledge about their local environment and that collaboration with scientific approaches would enhance their ability to manage disasters such as runaway fires and droughts. In their view, such a partnership would ultimately lead to safer communities, reduced environmental damage and increased resilience against hazards.

Overall, the findings indicate a strong willingness among local communities to embrace a complementary approach that merges IK and WK for DRR. Participants saw this integration to strengthen their preparedness, protect vulnerable groups and improve overall community resilience. Their perspectives reflect a pragmatic approach – one that values tradition while recognising the benefits of modern scientific advancements in ensuring sustainable and effective disaster management strategies.

## Discussion

### Indigenous indicators used to predict the different hazards and disasters in the study sites

The findings reveal that rural communities in Joe Morolong rely heavily on indigenous indicators to forecast seasonal changes, hazards and disasters, valuing these natural signs over modern weather alerts received via radio or television. Participants expressed strong confidence in these traditional methods, emphasising their reliability and long-term predictive accuracy. As one participant stated: ‘Tlholego ga e ake’ – ‘Nature never lies’. (Bothithong, FGD2, Both2M24)

### Birds indicators

Bird behaviour emerged as one of the primary forecasting tools as seen in Table 4. Participants enthusiastically shared how they have observed birds for generations to predict forthcoming seasons. One participant explained:

‘Gantsi re amogela dikgang ka sealemowa gore go tlile go nna le morwalela, mme ka tlwaelo re itse gore se ga se ne se diragala fa re bona nonyane e e bidiwang Mmamasilanoka. Re lekola kwa dinonyane tse di elemang teng mme se se tla re lemosa gore a go tla nna le merwalela kgotsa nnya? (Dithakong, FGD5, Dith4M43)

**TABLE 4:** Birds indicators.

Bird or animal name			Hazard predicted	Explanation of the indicator
Local name	English name	Scientific name		
*Dinonyana tše Ntsho (di metša)*	Black Birds (swallows)	*Hirundinidae*	Rain-Floods	The arrival of a large number of swallows is widely regarded as a positive sign, symbolising the anticipation of abundant rainfall. In many cases, their presence is closely followed by the formation of dark clouds, reinforcing their role as a natural indicator of impending rain.
*Tale*	Southern masked weaver	*Ploceus velatus*	Floods or drought	The nesting behaviour of this bird serves as a key environmental indicator of seasonal rainfall patterns. When the bird nests among the reeds in the valley, it signals the likelihood of poor or minimal rainfall, potentially leading to drought. Conversely, the absence of nests in the valley suggests an impending flood season, as the birds instinctively avoid areas prone to rising water levels.

The scientific names mentioned herein are confirmed by data obtained from the following sources: https://www.adda247.com/defence-jobs/scientific-names-of-animals/; https://www.vedantu.com/biology/scientific-name-of-goat, and Turner (2020).

Loosely translating to:

‘We always receive information on the radio that it is going to flood, but we always know that this will not be the case after observing the bird called Tale. We look at where these birds are nesting, and this tells us if we will have floods or not.’

The presence and behaviour of certain bird species, such as Tale birds and blackbirds, serve as indicators of flooding cycles or rainfall, allowing community members to take precautionary measures such as water harvesting, storage and preparing for potential floods. The presence of swallows, in particular, signals the arrival of heavy rains, prompting individuals to make necessary adjustments in anticipation of the changing weather.

### Lunar, climatic and wind indicators

Beyond birds, participants demonstrated extensive knowledge of lunar, climatic and wind indicators in Table 5 below. The moon, wind speed and direction and cloud formations are all closely monitored to predict upcoming environmental changes. The appearance of dark clouds, for example, is a reliable sign of excessive rainfall expected within hours. Meanwhile, the positioning of the moon is used to predict the quality of the rainy season. A participant explained:

‘Fa o ka re ngwedi kgotsa kgwedi ya wa, se se re bontsha gore go tile go nna le letlha le lentle la dipula tsa matsorotsoro, mme ka jalo re tshwanetse go ipaakanyetsa temo.’ (Bothithong, FGD4, Both4M28)

**TABLE 5:** Lunar, climatic and wind indicators.

Signs observed to predict hazard	Hazard predicted	Explanation of the sign
August rainfall	Drought	The absence of rainfall in August is a strong indicator of an impending drought season. Even if rain falls later in spring, it does not have the same impact as the critical August rains, which are essential for sustaining moisture levels in these areas.
Full moon	Drought	A full moon is a rare sight, and when it appears and lingers, it serves as a warning sign of an impending drought.
Falling (Descending) moon	Rain	A descending moon is often seen as a promising sign, indicating the likelihood of a good rainy season.
Black heavy clouds	Rain and hailstorm	The appearance and movement of dark, dense clouds often signal imminent rainfall, potentially within hours or the next few days. However, exceptionally dark clouds raise concern, as they indicate the likelihood of an approaching hailstorm.
Wind speed and direction	Rain	In these communities, a westward wind (Bophirima) is seen as a warning sign, often indicating that intense rainfall is approaching and potentially bringing hazardous conditions.
Extremely white clouds	Windstorm	The appearance of unusually white clouds, resembling ice, signals the approach of a windstorm. These clouds have long been associated with intense windstorms that can cause significant damage, especially to rooftops.
Extreme heat and wind	Floods	Persistent high heat levels that are accompanied by winds are indicators of severe rainfall, which may result in floods.

Loosely translating to:

‘When the moon looks like it is falling, it tells us that we are going to have a very good rainy season, and therefore we must prepare for planting’.

This knowledge enables farmers to make informed decisions about when to plant crops, reducing the risk of poor yields and ensuring agricultural success. Participants affirmed that careful observation of natural elements such as the sun, moon, wind and rain plays a crucial role in their daily decision-making. This knowledge allows them to determine the best times for activities such as grazing livestock, closing windows to protect against storms and planting crops at optimal periods. Participants involved in subsistence farming stressed that understanding these climate indicators is essential for avoiding crop damage and ensuring better compatibility between crops and weather conditions.

Wind indicators, in particular, help communities brace for storms and mitigate potential damage. Some participants reported placing stones on specific corners of their houses as a protective measure against strong winds that could displace roofs. Others described how they use trenches to divert water away from their homes during floods, effectively reducing the risk of water damage. In addition, the concept of *ubuntu* – which embodies communal support and solidarity – was highlighted as a key survival strategy during extreme weather events. As one participant described:

‘Mo nakong ya merwalela, re itshereletsa ka go tshabela mo dintlong tse tse di agilweng mo karololwaneng ya lefatshe e e kwa godingwana kgotsa mo leropong kgotsa ka go tiisa dipota ka maje go thibela metsi go tsena mo dintlong.’ (Tsineng, FGD3, Tsine3M73)

Loosely translating to:

‘During floods, we seek protection in homes built on higher ground or reinforce gabion walls made of rocks to keep water out of our homes’.

This demonstrates how IK not only informs early warning systems but also fosters a collective approach to disaster resilience.

Overall, these findings illustrate the deep trust that communities place in indigenous forecasting methods, which are based on generations of careful observation and adaptation to the natural environment. While modern weather predictions are acknowledged, they are often viewed as less reliable than the signs provided by nature. The use of bird behaviour, lunar and wind patterns and communal disaster response mechanisms demonstrates a sophisticated understanding of environmental changes, allowing communities to prepare for hazards effectively and maintain resilience in the face of disasters.

### Plants, shrubs and herbs indicators

In addition to birds, lunar, climatic and wind indicators, participants in the study sites also rely on plants and shrubs mentioned in Table 6 to predict drought conditions. Among the most significant indicators are the Mongana (blackthorn) and Moku (Vachelia) trees, which serve as crucial signals of impending environmental changes. Villagers closely observe the colour and condition of the leaves to forecast whether a drought is approaching.

**TABLE 6:** Plants and shrubs indicators.

Signs observed to predict hazard	Plants, shrubs, herbs name			Hazard predicted	Explanation of the indicator
	Local name	English name	Scientific name		
Colour and condition of shrub	*Mongana*	Blackthorn	*Acacia mellifera*	Drought	The Mongana shrub remains green year-round, even in winter and dry seasons. However, if its leaves turn yellow and dry out, it signals the onset of the drought season. When many of these trees begin to die, it indicates that the drought will be severe.
Colour and condition of shrub	*Moku*	Vachellia robusta	*Acacia robusta*	Drought	Similar to the Mongana, when the leaves of the Moku turn yellow and fall off, it signals an impending drought. If the Moku begins to dry out and turn black, it indicates the severity of the drought.

The scientific plant names mentioned herein are confirmed by data obtained from the South African National Biodiversity Institute (SANBI) website: https://pza.sanbi.org/leonotis-leonurus.

Throughout the FGDs, a common theme emerged: these plants provide a sense of foresight and preparation for what lies ahead. One Bendell FGD participant vividly captured this sentiment, pointing to a large tree in the distance and explaining:

‘A o bona setlhare se se tonna sele “Se phela se le tala ka nako tsotlhe mme fa se simolola go nna serolwana, re a bo re itse gore mathata a mo tseleng. Mme mathata a, a oketsegela go ya pele fa dimela di simolola go omelela le swa. Mo ngwageng oo, ga re kitla re lema ka ntlha ya fa temo e ikaegile ka pula.’ (Bendell, FGD1, Bend1M1)

Loosely translating to:

‘Can you see that big tree out there? It is always green throughout the year. But once its leaves start to turn yellow and fall off, we know that trouble is on the way. It is even worse if most of these plants start dying back. That year we will not even plough as we rely on the rain for our crops.’

This statement reflects the deep trust communities place in these natural indicators and their ability to guide agricultural and survival strategies.

These plants are widespread across the villages where the study was conducted, and participants reported that the practice of using them as indicators is still actively maintained. The vast majority of participants expressed confidence in their ability to interpret the conditions of Mongana and Moku trees to make informed decisions. This knowledge enables them to take pre-emptive measures such as planting drought-resistant crops, delaying planting altogether and ensuring sufficient water storage in traditional water barns. For livestock farmers, the sight of deteriorating trees serves as a signal to reduce their herds by selling off some animals before the drought intensifies.

Beyond individual actions, participants highlighted the importance of collective response mechanisms. When drought signs become evident, they regularly seek support from the *Kgosi* (traditional leadership), community representatives and government authorities. However, they emphasised that the responsibility of responding to drought ultimately falls on them. Their deep-rooted knowledge of plants, shrubs and herbs has empowered them to take proactive steps to safeguard their livelihoods. Rather than waiting for external intervention, they see themselves as the first line of defence against the devastating effects of drought, demonstrating how IK remains a vital tool in navigating environmental uncertainties.

### Environmental indicators

Focus group participants demonstrated a deep understanding of their environment, relying on specific environmental indicators shown in Table 7 to forecast hazardous events for the upcoming season. They identified soil conditions and borehole water levels as key indicators for predicting seasonal changes, much like their reliance on plants, shrubs and herbs. These indicators serve as a crucial guide for decision-making, allowing communities to take necessary precautions in anticipation of disasters. One Bothithong FGD participant described the role of soil conditions in predicting floods, stating:

‘Fa mmu o simolola go oma le go phanyega, re itse gore re tsena mo mo nakong ya leuba Mme fa mmu o phela o ikanye le go nna montsho re solofela dipula tse kgatlhisang.’ (Bothithong, FGD4, Both4M29)

**TABLE 7:** Environmental Indicators.

Signs observed to predict hazard	Hazard predicted	Explanation of the sign
Extreme dryness of the soil	Drought	Participants can predict the upcoming dry season by observing the soil’s condition. For these communities, extreme dryness and cracked ground are key signs of the approaching dry period. If this dryness continues, it often leads to a drought.
Boreholes	Drought	Villages rely on borehole water for both livestock and human consumption. When these boreholes dry up and no water flows, it signals the depletion of underground water, serving as a clear indicator of an impending drought.

Loosely translating to:

‘When the soil becomes too loose and cracks appear, we know that we are heading for a dry spell. But when the soil remains compact and dark, we can expect good rain.’

This knowledge enables them to act accordingly, ensuring that they are prepared for the challenges ahead.

In times when floods are expected, participants explained how they adjust their daily routines to enhance safety. One participant remarked:

‘Re tsibosa bana ba rona go nna mo magaeng le go tswa kwa mmileng ka ntlha ya gore go na le go sa nne le ponalopele ya go goroga ga merwalela mme e bile se se kotsi thata.’ (Gasese, FGD2, Gase2F16)

Loosely translating to:

‘We tell our children to stay indoors and off the streets because when the floods come, they are unpredictable and dangerous’.

Those living in low-lying areas proactively relocate to higher ground, staying there until the rains subside. Another common practice among participants involves constructing drainage trenches around their homes to divert water and prevent flooding. A participant from Dithakong FGD highlighted the importance of this, saying: ‘Re epa mesele ya metsi go dikologa dintlo go thibeletsa gore metsi a se tsenelele mo matlong ka ntlha ya fa se, se ka tlisa mathata a le mantis’ (Dithakong, FGD1, Dith1M30), loosely translating to: ‘We dig small channels so that the water does not collect around our houses, otherwise, it will start seeping in, and that brings all sorts of problems’.

For those engaged in animal husbandry, environmental indicators also inform their decisions. During flood-prone periods, shepherds opt to keep their livestock in barns or enclosures rather than allowing them to graze freely, minimising the risk of losing animals to sudden water surges. As one Bendell village participant puts it:

‘Fa re bona ditsiboso, re a itse gore re tshwanetse go baya diphologolo gaufi go na le go go ditlogela gore disasanke kgakala ka gore di ka nna tsa latlhega nako nngwe le nngwe.’ (Bendell, FGD5, Bend4M13)

Loosely translating to:

‘When we see the signs, we know that it’s better to keep our animals close rather than let them wander and risk losing them’.

Similarly, indicators of drought influence how livestock farmers plan for the dry season. If the signs suggest prolonged drought, those who can afford it begin stockpiling drought feed in advance to sustain their animals when grazing becomes scarce. A participant explained:

‘Ga re emele gore go nne le komelelo, mme fa re lemoga maemo a sa siamang a loapi, re reka dijo tsa diphologolo kgotsa luserene go sa le nako pele tlhwatlhwa ya tsone e ya kwa godimo.’ (Tsineng, FGD3, Tsine3M70)

Loosely translating to:

‘We don’t wait until the drought is upon us; if we see the signs, we buy feed early while it’s still affordable’.

These environmental indicators, deeply rooted in IK, continue to shape how communities prepare for and respond to natural hazards, allowing them to minimise risks and safeguard both their homes and livelihoods.

### Superstitions or rituals

Participants from Bendell spoke with great reverence about *Moroka wa pula*, or ‘rain queens’, individuals believed to possess extraordinary abilities to predict climatic conditions, particularly rainfall. These rain queens, they explained, do not rely on conventional meteorological methods but rather draw on their spiritual connection with higher powers and ancestral spirits to foresee whether rain will fall. Their role extends beyond prediction – they also perform sacred rituals to invoke rainfall when necessary. One participant, with a tone of deep conviction, shared how invaluable these figures have been in their lives:

‘Wa bona ngwanaka, dikgosigadi tsa pula di di re bolokile go le gontsi ka go re tsibosa ka dipula tse di tlang. Dikgosigadi tse, ba rerisana le badimo go bona fa re tlile go nna le pula kgotsa jang.’ (Bendell, FGD4, Bend4F4)

Loosely translating to:

‘You see, rain queens, my girl, they have saved us many times by warning us of the coming rains. They consult with our ancestors to see if we will have rain or not. This sentiment underscored the deep trust and reliance the community places in their abilities’.

The role of the rain queens is not just spiritual but also advisory. They serve as KIs to the royal house, ensuring that traditional leaders have the necessary foresight to prepare for the season ahead. By interpreting the messages they receive from ancestral spirits, they provide crucial insights into whether heavy rainfall is expected, thereby allowing leaders to disseminate timely warnings to the community. One participant reflected on their importance, emphasising how their wisdom has safeguarded the village in times of uncertainty: ‘Fa dikgosigadi tsa pula di bua, re a reetsa. Di lemosa baeteledipele ba rona, mme ka ntlha ya bona, re phela re ipaakantse’ (Bendell, FGD2, Bend2F3). ‘When the rain queens speak, we listen. They guide our leaders, and through them, we are prepared’. Their revered status within the community means that their words carry significant weight, and many residents continue to follow their guidance without question. This unwavering faith in the rain queens highlights the community’s deep-rooted belief in IKS, where spiritual insight and environmental awareness work hand in hand to mitigate potential disasters.

## Conclusion

For generations, indigenous communities have relied on their deep-rooted knowledge systems to predict, prepare for and endure hazardous events. Through careful observation of natural indicators such as birds, plants, the moon and wind patterns, they have developed a sophisticated understanding of environmental changes. This aligns with Hendry and Fitznor (2012) assertion that all human development is shaped by continuous interaction with the natural elements – soil, air, climate, plants and animals – which form the foundation of thriving communities. Similarly, Durie (2004) highlights the defining relationship between indigenous peoples and their land, forests, waterways and the atmosphere, which they depend upon for survival.

The findings of this study suggest that participants possess a deep awareness of both IKS and WKS. While the migration of knowledge across cultures and time is complex and dynamic (eds. Lackner, Amelung & Kurtz 2021), participants demonstrated an ability to distinguish between these two systems. They perceive IK as a body of wisdom derived from lived experiences, whereas WK is often seen as a product of technological advancement. This distinction reinforces the idea that knowledge is shaped by the accessibility and immediacy of information in one’s environment (ed. Sillitoe 2009). Participants consistently identified a range of indicators they use to predict disasters, including birds, plants, lunar phases, wind and other environmental signals. These indicators have long guided their preparedness, mitigation and response strategies. Participants expressed confidence in the reliability of their traditional forecasting methods, believing that their ability to anticipate hazardous events has been crucial for community resilience. A particularly fascinating aspect of this belief system is the role of spiritual figures such as rain queens, who are revered for their ability to predict rainfall and even perform rituals to invoke it. Their insights are highly valued, shaping decisions at both the household and community levels.

Given the steadfast trust that indigenous communities place in their knowledge systems, it is imperative for DRR practitioners to acknowledge and integrate these indicators into conventional weather forecasting methods. Participants emphasised that unless official forecasts align with their indigenous indicators, they are unlikely to take heed. This underscores the profound influence of IKS on decision-making, particularly in matters of disaster preparedness, livelihoods and even healthcare. The significance of such knowledge has been demonstrated globally, including its reported role in mitigating the impact of the 2004 Indian Ocean tsunami in Simeulue Island, Indonesia.

However, while IK remains an invaluable resource, the rapidly changing climate and the increasing frequency of extreme weather events present new challenges that require a more integrated approach. Sole reliance on traditional indicators may no longer be sufficient. Instead, a combined use of IK and WK could enhance the effectiveness of early warning systems and reduce disaster risks. Achieving this, however, will require significant public education and awareness campaigns to foster trust in both knowledge systems. Furthermore, meaningful collaboration between researchers, practitioners and indigenous communities is essential in finding common ground for disaster risk management.

It is important to note that the findings of this study are drawn from a limited sample of participants and may not be representative of the entire population within the study area. As qualitative research prioritises depth over breadth, the intent is not to generalise but to provide context-specific insights. Indigenous communities are diverse, and their knowledge systems are shaped by unique environmental and cultural contexts. Nevertheless, this study provides compelling evidence of the need for a complementary approach that integrates both IKS and WKS in DRR. Future research should further explore how these knowledge systems can be harmonised to enhance community resilience in the face of evolving climatic challenges.
